# Heavy Metals in Follicular Fluid Affect the Ultrastructure of the Human Mature Cumulus-Oocyte Complex

**DOI:** 10.3390/cells12212577

**Published:** 2023-11-05

**Authors:** Selenia Miglietta, Loredana Cristiano, Ezio Battaglione, Guido Macchiarelli, Stefania Annarita Nottola, Maria Paola De Marco, Flavia Costanzi, Mauro Schimberni, Nicola Colacurci, Donatella Caserta, Giuseppe Familiari

**Affiliations:** 1 Department of Anatomy, Histology, Forensic Medicine and Orthopaedics, Sapienza University, 00165 Rome, Italy; ezio.battaglione@uniroma1.it (E.B.); stefania.nottola@uniroma1.it (S.A.N.); giuseppe.familiari@uniroma1.it (G.F.); 2 Department of Life Health and Environmental Sciences, University of L’Aquila, 67100 L’Aquila, Italy; loredana.cristiano@univaq.it (L.C.); guido.macchiarelli@univaq.it (G.M.); 3 Department of Medical and Surgical Sciences and Translational Medicine, Sapienza University of Rome, Sant’Andrea University Hospital, Via di Grottarossa 1035, 00189 Rome, Italy; demarco.mariapaola@gmail.com (M.P.D.M.); costanzi.flavia@gmail.com (F.C.); donatella.caserta@uniroma1.it (D.C.); 4 GENERA Centers for Reproductive Medicine, Clinica Valle Giulia, 00197 Rome, Italy; bioroma@bioroma.net; 5 Department of Woman Child and General and Specialized Surgery, University of Campania “Luigi Vanvitelli”, 80138 Naples, Italy; nicola.colacurci@yahoo.it

**Keywords:** heavy metals, lead, cadmium, oocyte, cumulus cells, human, electron microscopy

## Abstract

**Highlights:**

**What are the main findings?**

- For the first time, ultrastructural alterations were detected using transmission electron microscopy in human cumulus-oocyte complexes (COCs) sampled from follicles with Pb and Cd levels in the FF of infertile women subjected to assisted reproductive technologies.
- The intrafollicular presence of these metals could be responsible for morphological alterations in some cell organelles, which may lead to altered maturation and quality of oocytes, impairment of energetic metabolism, cellular dysfunction, and apoptosis of CCs.
- Since blood Cd levels were above the current reference values established by the Agency for Toxic Substances and Disease Registry (ATSDR) and the Environmental Protection Agency (EPA), whereas blood Pb levels were below the ATSDR reference values, we believe that these alterations could be due especially to Cd, even if we cannot exclude a possible additional effect of Pb.

**What is the implication of the main finding?**

- Cd levels in FF may influence the oocyte directly and indirectly (CC-mediated), affecting its quality, as well as fertilization, early embryonic development, and pregnancy.

**Abstract:**

It is known that exposure to heavy metal such as lead (Pb) and cadmium (Cd) has several adverse effects, particularly on the human reproductive system. Pb and Cd have been associated with infertility in both men and women. In pregnant women, they have been associated with spontaneous abortion, preterm birth, and impairment of the development of the fetus. Since these heavy metals come from both natural and anthropogenic activities and their harmful effects have been observed even at low levels of exposure, exposure to them remains a public health issue, especially for the reproductive system. Given this, the present study aimed to investigate the potential reproductive effects of Pb and Cd levels in the follicular fluid (FF) of infertile women and non-smokers exposed to heavy metals for professional reasons or as a result of living in rural areas near landfills and waste disposal areas in order to correlate the intrafollicular presence of these metals with possible alterations in the ultrastructure of human cumulus-oocyte complexes (COCs), which are probably responsible for infertility. Blood and FF metals were measured using atomic absorption spectrometry. COCs corresponding to each FF analyzed were subjected to ultrastructural analyses using transmission electron microscopy. We demonstrated for the first time that intrafollicular levels of Pb (0.66 µg/dL–0.85 µg/dL) and Cd (0.26 µg/L–0.41 µg/L) could be associated with morphological alterations of both the oocyte and cumulus cells’ (CCs) ultrastructure. Since blood Cd levels (0.54 µg/L–1.87 µg/L) were above the current reference values established by the guidelines of the Agency for Toxic Substances and Disease Registry (ATSDR) and the Environmental Protection Agency (EPA) (0.4 µg/L), whereas blood Pb levels (1.28 µg/dL–3.98 µg/dL) were below the ATSDR reference values (≤5 µg/dL), we believe that these alterations could be due especially to Cd, even if we cannot exclude a possible additional effect of Pb. Our results highlighted that oocytes were affected in maturation and quality, whereas CCs showed scarcely active steroidogenic elements. Regressing CCs, with cytoplasmic alterations, were also numerous. According to Cd’s endocrine-disrupting activity, the poor steroidogenic activity of CCs might correlate with delayed oocyte cytoplasmic maturation. So, we conclude that levels of heavy metals in the blood and the FF might negatively affect fertilization, embryo development, and pregnancy, compromising oocyte competence in fertilization both directly and indirectly, impairing CC steroidogenic activity, and inducing CC apoptosis.

## 1. Introduction

The word ‘heavy metal’ has been used to describe metallic chemical elements and metalloids that are toxic to the environment and humans [1]. The main source of heavy metal (HM) pollution is primarily the mining of metal, smelting, foundries, and other metal-based industries. Heavy metal use in agriculture represents the secondary source of heavy metal pollution (use of pesticides, insecticides, and fertilizers). HMs, widely used in industry, agriculture, medicine, and other sectors, are dispersed into the environment, including our atmosphere, waters, and soils; they may enter the human body by ingesting contaminated food, inhalation, drinking contaminated water, and skin contact. They are classified as dangerous, and their bioaccumulation causes biological and physiological complications [1]; inside the reproductive tissues of both women and men, they cause harmful effects by interfering with normal cellular metabolism and the correct performance of vital functions [2,3,4]. Cellular organelles such as mitochondria, nuclei, lysosomes, and membranes have been reported to be affected by HMs. At the nuclear level, they are recognized for their carcinogenic properties since they interact with nuclear proteins and may cause both conformal changes and inactivation of molecules (direct damage) and free radicals’ production (indirect damage), resulting in DNA damage, disruption of antioxidant defenses, and lipid peroxidation [5,6]. HMs may interfere with both lipids, causing cell membrane damage and impairment of calcium homeostasis, and proteins (misfolding, aggregation, and conformational change enzyme activation), causing cellular function loss [1]. In addition, some HMs may affect the synthesis of estrogen and progesterone in women, causing related hormonal metabolic disease [7]. 

Cd and Pb are the most toxic HMs, and are known for their adverse effects, particularly on the human reproductive system [8]. Pb exposure is associated with menstrual cycle disorders, preterm birth, and miscarriage, and, in pregnant women, lead can cross the placenta and impair the development of the fetus. Therefore, exposed women are at risk of spontaneous abortion, premature delivery, gestational diabetes mellitus, pregnancy hypertension, preeclampsia, premature rupture of membranes, intrauterine growth restriction, low-weight birth, and other pregnancy complications [9]. Cd has a hazardous effect on sperm quality and natural embryo development [10,11]. In addition, Cd exposure disturbs follicle development, causing developmental disorders of primordial follicles and increasing the number of atretic follicles [10]. High levels of Cd in FF impair the oocyte quality [8]. In mammals, Cd exposure impairs oocyte meiotic maturation rate both in vivo and in vitro. It alters the quality of oocytes via oxidative stress, leading to a decrease in female fertility [12,13]. Cd has been correlated with an increased risk of breast and endometrial cancer, endometriosis, and infertility, especially at high doses and occupational-level exposures, and has a harmful effect on assisted reproductive technology (ART) outcome, on the grading of embryo quality and on embryo transfer (ET) [14,15,16]. Low-level environmental exposure to Pb and Cd may interfere with pregnancy during in vitro fertilization [7].

Given this, the present study aimed to investigate the presence of Pb and Cd in the FF of women occupationally exposed to these metals and subjected to ART procedures (intracytoplasmic sperm injection, ICSI, or in vitro fertilization, IVF) to correlate FF levels of these metals with possible alterations in the ultrastructure of human COCs responsible for the outcome of pregnancy. 

## 2. Materials and Methods

### 2.1. Ethics Statement

The study was conducted in accordance with the Declaration of Helsinki and approved by the Ethics Committee of the Department of Surgical and Medical Sciences and Translational Medicine of Sant’Andrea University Hospital, Sapienza University of Rome (Protocol No. 9384) for studies involving humans, and all patients agreed to participate in the study and signed a written informed consent form for all procedures to be carried out.

### 2.2. Study Population

In total, 17 couples undergoing ART procedures (ICSI and FIVET) at the reproduction unit of GENERA in Rome, Italy, were recruited for our study from October 2017 to June 2019, based on the following criteria: (1) females aged between 24 and 39 years; diagnosed infertility (failure to achieve a pregnancy after 12 months or more of regular unprotected sexual intercourse) due to unexplained infertility; tubal factors; and male factor (poor semen quantity and quality) (Table 1). A detailed questionnaire collected demographic, lifestyle, reproductive, and medical history information. All women were non-smokers but were exposed to HMs for professional reasons (garbage women and crossing guards) or as a result of living in rural areas near landfills and waste disposal areas.

### 2.3. Sample Collection

#### 2.3.1. FF and COCs Sampling

Women initiating IVF treatment underwent gonadotropin-induced ovarian stimulation according to standard clinical protocols. Follicular maturation and endometrial development were monitored by measuring serum estradiol levels and transvaginal ultrasound. The hCG was administered when enough follicles ≥17 mm in diameter developed. Oocytes were retrieved using transvaginal fine needle aspiration within 36 h of hCG administration. FFs were collected from the two largest individual follicles in each ovary (up to four follicles per woman) following recovery of the oocyte for clinical use. We have selected two oocytes for an excellent guarantee of effective detection. Three/four follicles were collected for most patients (82%), while three (18%) women contributed only two. From a single individual follicle, with an average follicular diameter of 18–21 mm, an FF volume of 3–4 mL was collected using a fresh needle. A new needle was used for each woman, and aspiration needles were rinsed with saline before each individual puncture. However, the follicle itself was not rinsed. For each patient, collected FFs were then placed in sterile metal-free 1.5 mL microcentrifuge tubes (Labcon, Petaluma, CA, USA), examined for evidence of blood contamination [17], centrifuged, and divided into aliquots before freezing at −80 °C pending analysis. A wash buffer or contaminant was not used in our samples [18]. Oocytes obtained from ovarian stimulation were inseminated to produce a maximum of three embryos (Table 1) and then transferred into the uterus. Supernumerary oocytes (1–3/woman), corresponding with respective aspired FF, surrounded by intact CCs with homogeneous cytoplasm, were subjected to ultrastructural analyses according to Miglietta et al. (2023) [19].

#### 2.3.2. Cd Detection Using Atomic Absorption Spectrometry

Specimens of FF from each woman were analyzed for the determination of trace Cd elements using graphite furnace atomic absorption spectrometry (AAS) (Applied Biosystems, Foster City, CA, USA) (Table 2). In this technique, the detection limits are excellent, and it is possible to reach picogram levels under favorable conditions. The samples are generally injected into the graphite cuvette as solutions ranging from 10 to 100 μL. The atomization is carried out at high temperatures, producing a collection of gaseous atoms exposed to the radiation of hollow cathode or electrodeless discharge lamps. The background correction is based on the Zeeman effect [20].

Taking into account the toxicological profiles for Pb and Cd established by guidelines of ATSDR and EPA (0.4 µg/L and ≤5 µg/dL, respectively) [21,22], the study population was divided into two groups: patients with low levels of Cd in the blood (0.54 µg/L–0.74 µg/L) and FF (0.10 µg/L–0.25 µg/L) (Group 1, 10 women/17) and patients with high levels of Cd in the blood (0.77 µg/L–1.87 µg/L) and FF (0.26 µg/L–0.41 µg/L) (Group 2, 7 women/17). However, in all patients, Pb values were lower than minimal health risk levels both in the blood (1.28 µg/dL–3.98 µg/dL) and in FF (0.49 µg/dL–0.85 µg/dL).

#### 2.3.3. Statistical Analyses

Statistical analysis was performed using MedCalc© Statistical software (version 20.218, MedCalc Software Ltd., Ostend, Belgium). Parametric (normally distributed) variables were expressed as mean ± standard deviation. To analyze the relationship between Pb and Cd concentrations in the blood and the FF, logistic regression analysis was used. The regression equation and analysis of variance were calculated. The 95% prediction interval and the 95% confidence interval were also calculated. Differences with *p* < 0.05 were considered statistically significant.

### 2.4. Electron Microscopy

COCs were fixed with 2.5% glutaraldehyde (Electron Microscopy Sciences, EMS, Hatfield, PA, USA) in phosphate-buffered saline solution (PBS) for at least 48 h at 4 °C. Fixed samples, after several washing in PBS, were post-fixed with a 1% osmium tetroxide (EMS) for 2 h, rinsed several times in PBS and embedded in agar 1% (EMS). Oocytes were then rinsed in PBS, dehydrated through an ascending series of ethanol, immersed in propylene oxide (Sigma-Aldrich, St. Louis, MO, USA) for 40 min, and left overnight in a propylene oxide/resin 1:1 solution. Finally, they were embedded in epoxy resin (Agar Scientific, Stansted, UK) for 48 h at 60 °C. Resin blocks were cut with a diamond knife (90–100 nm) using an Ultracut E ultramicrotome (Leica EMUC6, Wetzlar, Germany), and ultrathin sections were mounted on 100-mesh copper grids (Assing, Rome, Italy). Finally, sections were contrasted using Uranyless (Uranyl acetate alternative) (TAAB Laboratories Equipment Ltd., Aldermaston, UK) and lead citrate (Electron Microscopy Sciences) and analyzed using a TEM (Zeiss EM10, Oberkochen, Germany), operating at 60 kV. Images were acquired using a digital camera (AMT CCD, Deben UK Ltd., Suffolk, UK).

## 3. Results

### 3.1. Characteristics of the Study Population and Heavy Metal’s Measurements

Table 1 reported the demographic and clinical characteristics of the study population. A total of 17 couples undergoing ART procedures were recruited in this study. Female partners had an average age of 32.4 ± 4.4 years. At the couple level, 59% of couples had a diagnosis of a female factor alone, 35% had a diagnosis of a male factor alone, and 6% were diagnosed with unexplained infertility. The specific infertility causes are reported in Table 1. 

Regarding the outcomes, only 17.65% of women had term pregnancies. Women belonging to Group 1 had a pregnancy rate of 40% (4/10), with an abortion rate of 25% (1/4), and no pregnancy rate of 60% (6/10). Women in Group 2 had a pregnancy rate of 14% (1/7), with an abortion rate of 100% (1/1), and no pregnancy rate of 86% (6/7).

Since blood Pb and Cd levels represent a useful indicator of the degree of exposure in recent months, their presence was used for occupational biomonitoring of our study population.

Table 2 reported Pb and Cd levels in the blood and follicular fluid of the women under study. The range detection values for Pb in the blood of patients went from 1.28 µg/dL to 3.98 µg/dL with an average value of 2.58 µg/dL, resulting in much lower values than the minimal health risk levels (≤10 µg/dL) established using the guidelines developed by both the ATSDR and the EPA [22]. Our Pb values were, however, also lower than both the recommended blood lead concentration (5 µg/dL) detailed in The World Health Organization (WHO) guideline for clinical management of exposure to lead [23] and the reference values of blood Pb in women aged 18–69 (7 µg/dL) derived following the biomonitoring of the Italian population to heavy metal exposition [24].

Regarding the range detection values for Cd in the blood, we found values ranging from 0.54 µg/L to 1.87 µg/L with an average value of 0.82 µg/L. The guidelines ATSDR established state that 0.4 µg/L (CDC 2005) is the 95% confidence limit for blood cadmium levels in the United States for healthy nonexposed, non-smokers, and occupationally exposed persons who may have higher blood levels than the general population. 

**Figure 1:**
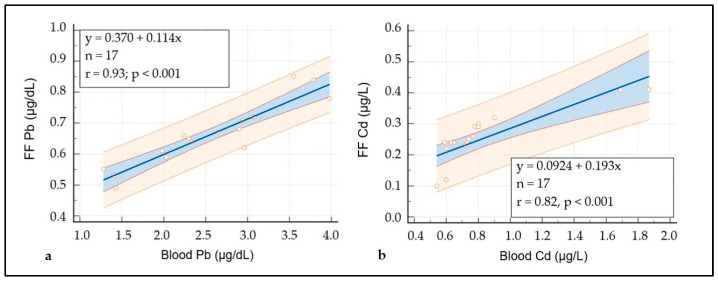
Degree of the relationship between blood Pb/cd and FF Pb/Cd levels. (**a**) Regression line for Pb (95% CI 0.81 to 0.97). (**b**) Regression line for Cd (95% CI 0.53 to 0.93). A 95% confidence interval and a 95% prediction interval.

The presence of Pb and Cd in samples of FF, with an average value of 0.67 µg/dL ± 0.06 μg/dL for Pb and 0.25 µg/L ± 0.01 µg/L for Cd (Table 2) indicated that the intrafollicular levels of the two metals were positively correlated to blood levels as demonstrated using the diagrams calculated using regression analysis (Figure 1a,b).

Based on these results, we divided the study population into two groups: patients with low levels of Cd in the blood (0.54 µg/L–0.74 µg/L) and the FF (0.10 µg/L–0.25 µg/L) (10 patients out of 17, corresponding to 59%) and patient with high levels of Cd in the blood (0.77 µg/L–1.87 µg/L) and the FF (0.26 µg/L–0.41 µg/L) (7 patients out of 17, corresponding to 41%). Interestingly, patients with high Cd levels also had higher Pb levels in the blood (2.65 µg/dL–3.98 µg/dL) and in the FF (0.66 µg/dL–0.85 µg/dL), although these values were lower than minimal health risk levels. 

### 3.2. Electron Microscopy

The ultrastructural analysis of human COCs using TEM demonstrated that Group 1 patients had a normal structure of COCs compared to those in Group 2, in which COCs showed anomalies. 

**Figure 2:**
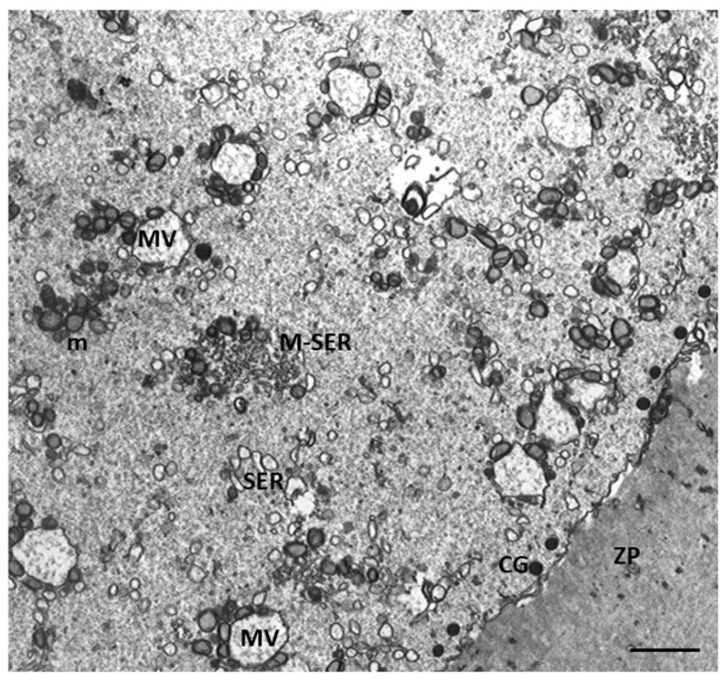
A representative picture of Group 1 oocytes’ cytoplasm ultrastructure.Mitochondria (m) are associated with the endoplasmic reticulum to form typical large, abundant M-SER aggregates and numerous MV complexes of variable dimensions. CG, cortical granules, ZP, zona pellucida. Bar = 1 µm.

Figure 2 shows ultrastructural cytoplasmic features of the oocytes of Group 1 patients that include the presence of cortical granules beneath the oolemma, and typical associations between tubular elements of smooth endoplasmic reticulum (SER) and mitochondria (m), called M-SER aggregates, interspersed in the cytoplasm among numerous vesicles of SER and mitochondria (MV complexes) of various sizes. 

**Figure 3:**
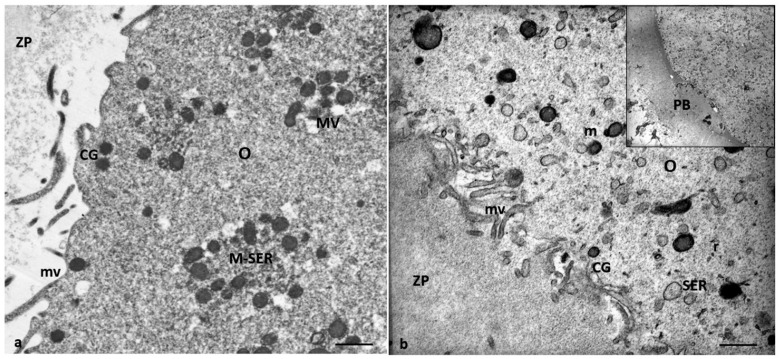
A representative picture of the cytoplasm of the oocytes of Group 1 and 2 patients. (**a**) Oocytes of Group 1 patients had a typical cytoplasm with mitochondria associated with SER cisternae (M-SER) and surrounding SER vesicles (MV). (**b**) Oocytes of Group 2 patients exhibit an immature cytoplasm (O) even if ((**b**), inset) the polar body (PB) is present. Isolated mitochondria (m), underdeveloped SER as aggregates of tubules, small vesicles, or isolated tubules, and numerous free ribosomes (r) are detected. ZP, zona pellucida; mv, microvilli. Bar = 800 nm.

Characteristics of cytoplasmic immaturity (Figure 3) were, instead, detected in the oocytes of Group 2 patients, even if the presence of the polar body demonstrated (Figure 3b, inset) the nuclear maturation. In contrast to the oocytes of Group 1 patients (Figure 3a), the oocytes of Group 2 patients showed a few cortical granules under oolemma, underdeveloped SER, either as aggregates of tubules or small vesicles, isolated mitochondria, not associated with SER tubules or vesicles (M-SER and MV), and free ribosomes sparsely present in the ooplasm (Figure 3b). 

**Figure 4:**
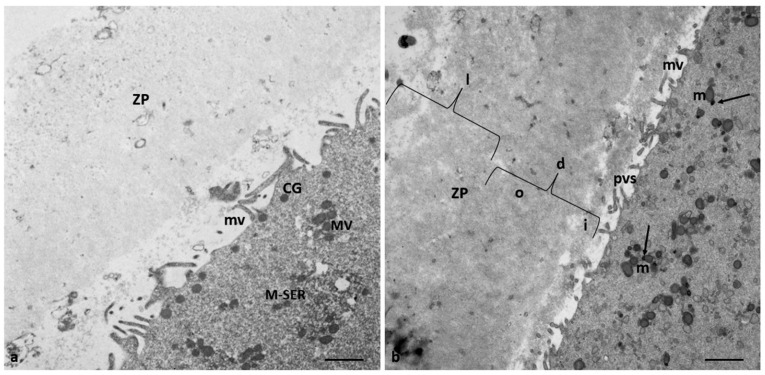
ZP comparison of Groups 1 and 2 patients. (**a**) The oocytes of Group 1 patients showed a ZP with a typical loose, microfilamentous architecture. (**b**) The oocytes of Group 2 patients exhibit a ZP composed of two regions with different densities: an outer area with a loose filamentous texture (l) and an inner area denser and more compact (d). In addition, the denser ZP area took on a particular bilayered structure characterized by a thick outer (o) portion and a thin inner portion (i), perivitelline space (pvs), and microvilli (mv). In contrast to the oocytes of Group 1 patients, characterized by M-SER aggregates and MV complexes (**a**), the oocytes of Group 2 patients present isolated mitochondria with prominent electron-dense granules (arrows) and minute SER vesicles. Also, cortical granules (CG) are absent. Bar = 1 µm.

In addition, in 57% of the oocytes of Group 2 patients (4/7), the zona pellucida (ZP) was also affected (Figure 4). In contrast to the oocytes of Group 1 patients (Figure 4a), the ZP exhibited two regions with different densities throughout its thickness: an outer area with a loose filamentous texture and a denser and more compact inner area. In addition, the denser ZP area took on a particular bilayered structure characterized by a thick outer portion and a thin inner portion (Figure 4b). However, there was no change in the whole ZP thickness, which was approximately 10–12 µm.

**Figure 5:**
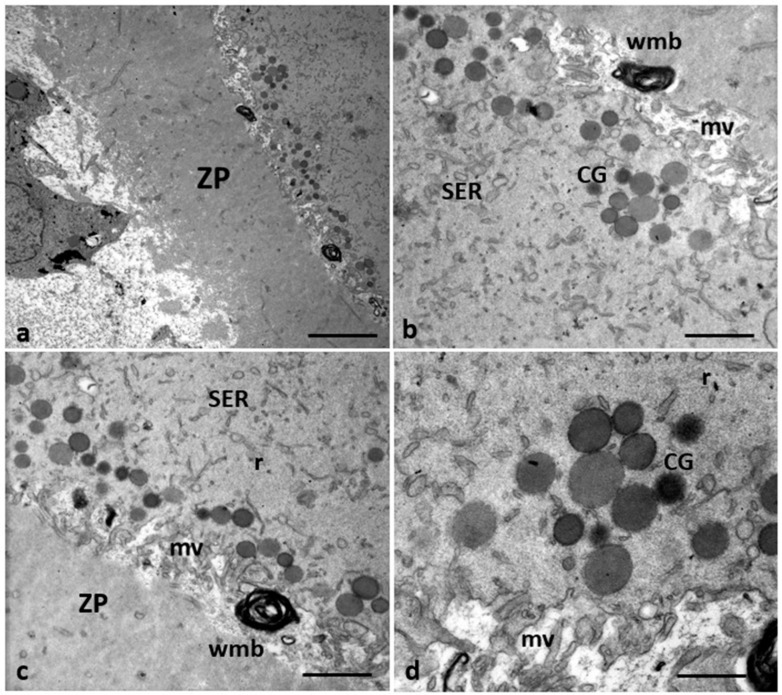
Altered distribution pattern of CG in the oocytes in Group 2 patients. (**a**) Low magnification of ZP and oocyte cortical area and (**b**,**c**) details at higher magnification of the cortical area with CG clusters; in the perivitelline space (pvs) whorled membranous bodies (wmb) were detected. Bar = 1.6 µm (**a**); Bar = 800 nm (**b**,**c**); Bar = 300 nm (**d**).

The remaining 43% (3/7) of the oocytes of Group 2 patients showed a typical microfilamentous architecture of the ZP and were provided by a proper amount of CGs with an altered distribution pattern (Figure 5). CGs formed a very discontinuous rim (Figure 5a,c), being grouped in clusters in the oocyte cortex (Figure 5b,d). Whorled membranous bodies were found in perivitelline space, probably derived from involuting altered microvilli (Figure 5b,c). 

**Figure 6:**
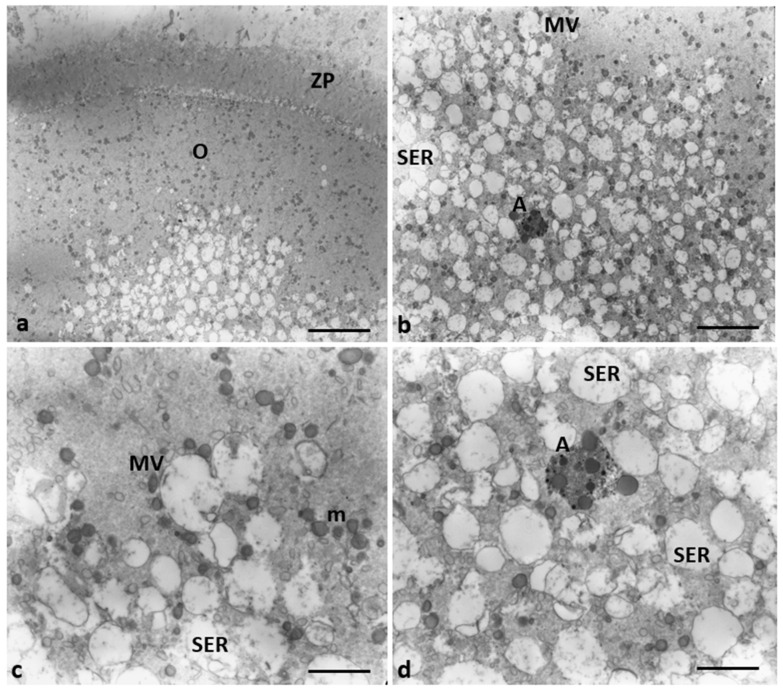
Evidence of endoplasmic reticulum stress in the oocytes of Group 2 patients. (**a**,**b**) Large, communicating dilated SER vesicles occur in the center of cytoplasm and (**c**,**d**) details at high magnification of scarce and large MV complexes and autophagosome (A) between SER vesicles that appear more numerous. Bar = 2 µm (**a**); Bar = 1 µm (**b**); Bar = 800 nm (**c**,**d**).

However, all the oocytes of Group 2 patients exhibit ultrastructural changes in some organelles. Figure 6 shows the presence of several large, communicating, dilated SER vesicles in the center of the ooplasm, with a secretory, moderately electrodense material in the lumen indicating endoplasmic reticulum stress leading to frank vacuolization. The presence of autophagosomes dispersed among the SER vesicles and large but scarce MV complexes sometimes showing interrupted membranes confirms possible metabolic cell suffering. 

**Figure 7:**
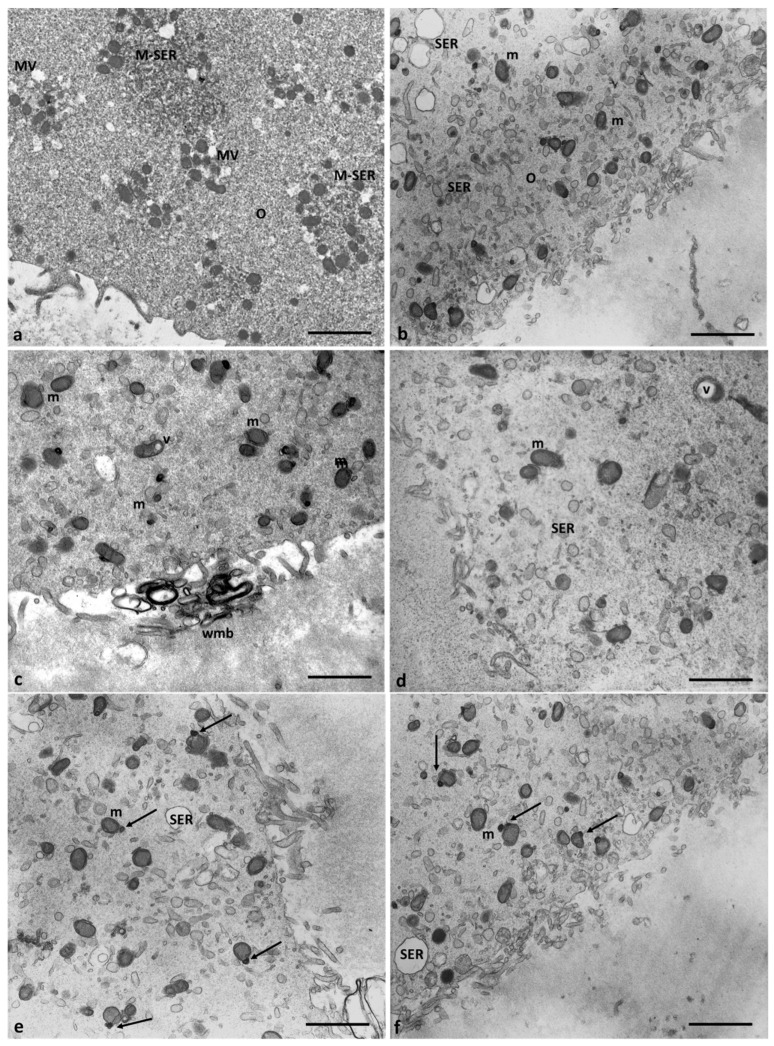
Mitochondria ultrastructural changes in oocytes of Group 2 patients. (**a**) Mitochondria of the oocytes of Group 1 patients are organized in M-SER aggregates and MV complexes. (**b**–**d**) Mitochondria of the oocytes of Group 2 patients appear isolated, are not associated with SER vesicles, and are both swollen with lapsed cristae and smaller/pycnotic; vacuolated (v) mitochondria are also present. (**e**,**f**). Note the mitochondrial matrix with prominent electron-dense granules (arrows). Bar = 1 µm.

In the oocytes of Group 2 patients, mitochondria were numerous and uniformly distributed. However, in association with the dilated endoplasmic reticulum, which was different from the control oocytes (Figure 7a), electrodense mitochondria with lapsed cristae and a denser matrix, and smaller, pycnotic, and electrodense ones were present in the cortical area (Figure 7b–d). Most mitochondria, dispersed in the cytoplasm and separated from SER vesicles, had a mitochondrial matrix containing prominent electron-dense granules (Figure 7e,f, arrows). Moreover, some vacuolated mitochondria have been observed. 

Since CCs closely interact with the oocyte not only after natural ovulation or follicle aspiration but also during and after fertilization, influencing various changes necessary for implantation and early development of the zygote, the study of possible Cd-induced morphological alterations was extended to these cells. A distinctive characteristic of CCs is their capacity to change from a compact cell mass around the oocyte into a dispersed structure of cells during the preovulatory period, a process called cumulus expansion that involves the synthesis and deposition of a mucoid fibrillar intercellular matrix. 

The ultrastructural analysis was focused only on the corona cells and clusters of the outer CCs, although these were slightly reduced in number due to unavoidable mechanical manipulation of the COCs [25,26].

**Figure 8:**
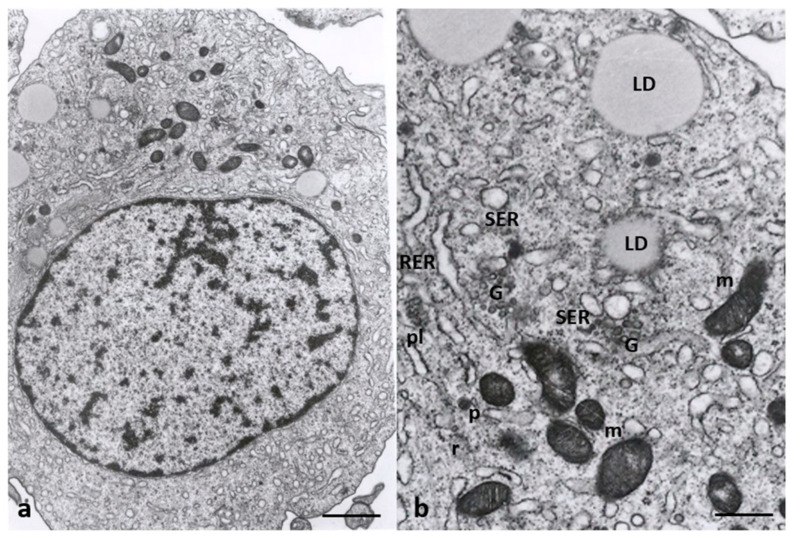
Cumulus cell ultrastructure of Group 1 patients. (**a**) Overview of the cell with a large, eccentric, and oval nucleus and numerous cytoplasmic organelles. (**b**) Detail of different types of organelles in the cytoplasm. LD, lipid droplets; SER, smooth endoplasmic reticulum; RER, rough endoplasmic reticulum; G, Golgi apparatus; m, mitochondria; r, ribosomes; pl, polyribosomes; p, micro-peroxisome. Bar = 30 µm (**a**); Bar = 12 µm (**b**).

Figure 8 shows the cytoplasmatic ultrastructure of CCs of Group 1 patients, attesting to the typical steroidogenic features of these cells. CCs appeared irregularly rounded, with an oval nucleus eccentrically located showing dispersed chromatin (Figure 8a); they also contained many types of organelles such as mitochondria with lamellar or tubular cristae, abundant membranes of SER, microtubules, microfilaments in close contact with SER elements, and abundant lipid droplets. The latter were also associated with mitochondria and SER membranes. Micro-peroxisomes, cisternae of rough endoplasmic reticulum (RER), ribosomes, vesicles, membranes belonging to the Golgi complex, and polyribosomes were also present in the cytoplasm (Figure 8b).

**Figure 9:**
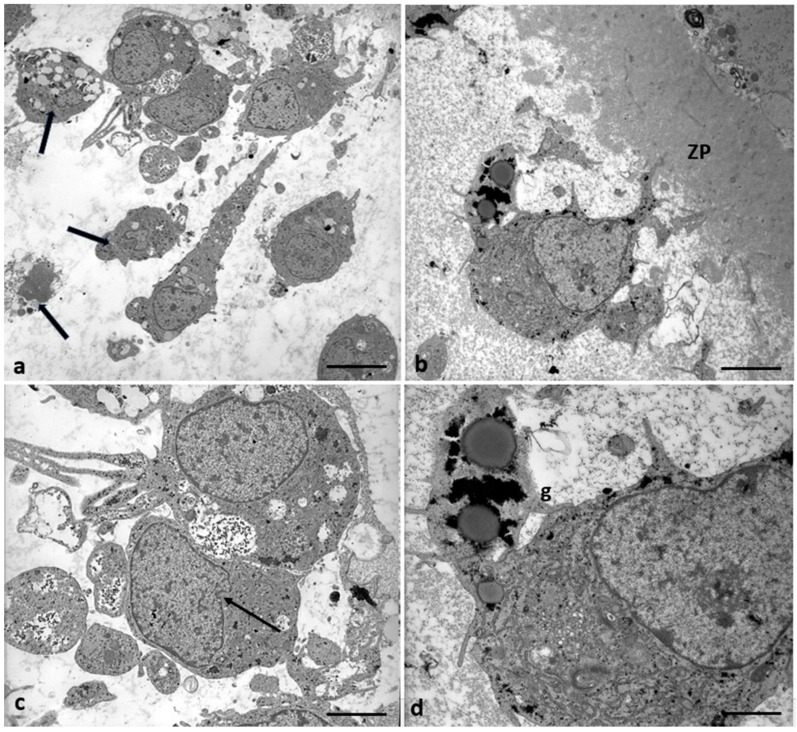
Morphological changes in the shape of CCs of Group 2 patients. (**a**) CCs dispersed within the extracellular matrix, with a shape irregularly rounded, polyhedral, or elongated, and some cells showing characteristic signs of apoptosis (thick arrows). (**b**) CCs with large cytoplasmic processes projected in the ZP. (**c**) cell nuclei appear flattened and indented (thin arrows), and (**d**) abundant granules (g) of glycogen appear dispersed in the cytoplasm, around lipid droplets, and inside vacuoles. Bar = 180 µm (**a**); Bar = 90 µm (**b**); Bar = 70 µm (**c**); Bar = 40 µm (**d**).

**Figure 10:**
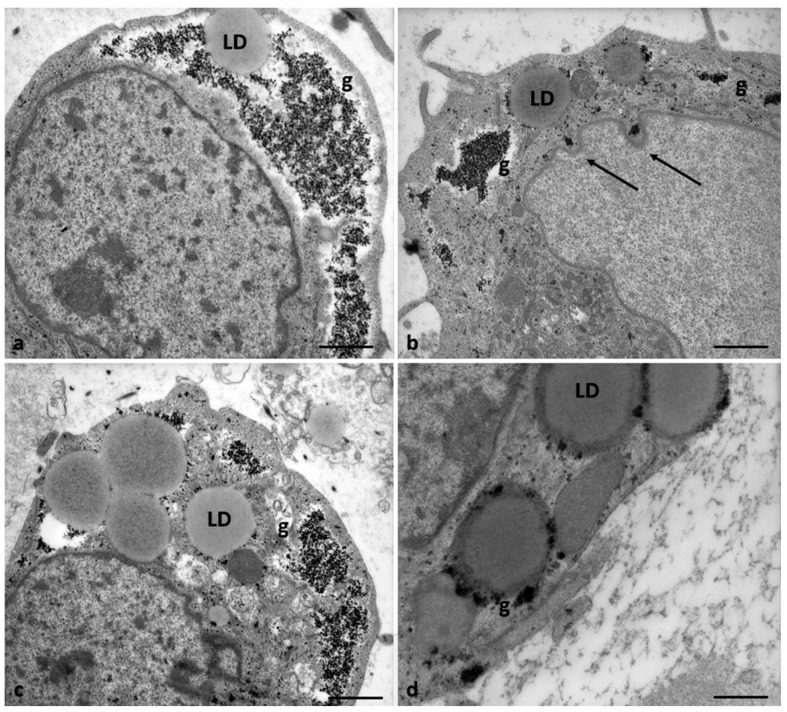
Accumulation of glycogen in CCs of Group 2 patients. (**a**,**b**) Abundant granules of glycogen (g) appear dispersed in the cytoplasm and (**b**,**c**) around lipid droplets (LD). Bar = 25 µm (**a**–**c**); Bar =18 µm (**d**).

In 71.4% of the Group 2 patients (5/7), the CCs appear with moderate electron density, dispersed within a fine and delicate fibrillar extracellular matrix, irregularly rounded, polyhedral, or elongated in shape (Figure 9a), with large and short cytoplasmic processes projecting through the ZP that did not seem to reach and contact oocyte microvilli (Figure 9b). The shape of nuclei changed from oval to flattened and indented (Figure 9c, thin arrow, and Figure 10b), and abundant glycogen granules were detected, dispersed in the cytoplasm, and around numerous and large lipid droplets. Micro-peroxisomes, organelles that confer the steroidogenic ability to these cells, have not been observed. 

**Figure 11:**
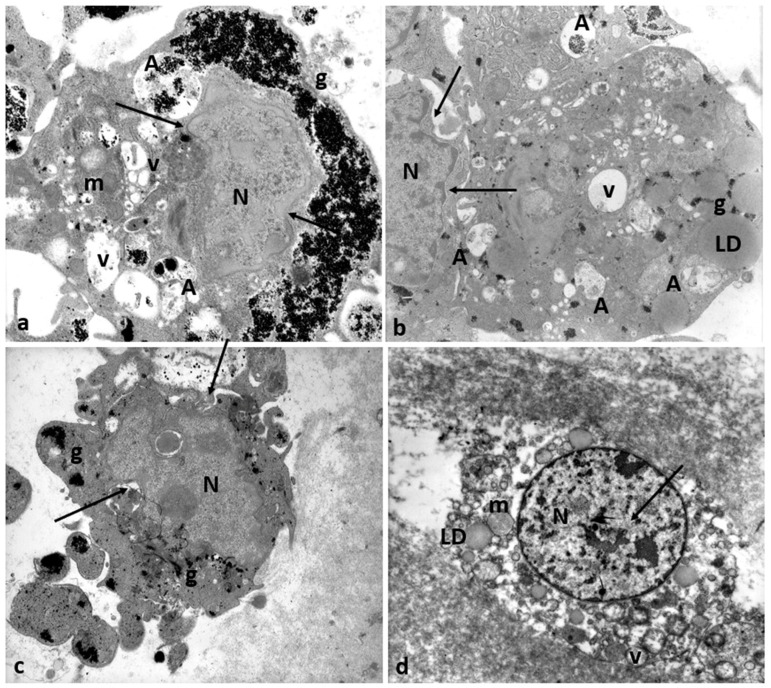
Regressing and degenerating CCs in Group 2 patients. (**a**–**c**) Numerous vacuoles (V) and autophagosomes (A) containing involuting organelles occur in the cytoplasm. Note the presence of abundant glycogen granules (g) peripherally accumulating around the nucleus (N) or inside vacuoles, cytoplasmic fragments, and cellular blebs. (**d**) Evidence of cytoplasmic lysis and vacuolization. Nuclear alterations occur (arrows). m, mitochondria; LD, lipid droplets. Bar = 35 µm.

In 28.6% of the Group 2 patients (2/7), CCs showed even characteristic apoptotic signs (Figure 9a, tick arrows, and Figure 11).

Figure 10 highlights details of the presence of abundant glycogen granules in the CCs of Group 2 patients. They were distributed particularly in the cytoplasm, close to nuclear indentations (Figure 10a,b, arrows), around lipid droplets (Figure 10c,d), which indicates a possible impairment of the metabolism of carbohydrates and glucose homeostasis. 

Figure 11 shows degenerating CCs in Group 2 patients. Numerous regressing elements such as vacuoles of different sizes and autophagosomes containing glycogen granules and involuting mitochondria are present (Figure 11a–c); cytoplasmic fragments deriving from cellular blebbing (Figure 11c), pyknotic (Figure 11c) or regressing nuclei (Figure 11a–c), and a dense or lysed, vacuolized cytoplasm (Figure 11d) were observed.

## 4. Discussion

It is now known that various exogenous environmental pollutants can influence female reproductive health and pre- and postnatal development, and few previous studies reported to date in females have shown a significant relationship between heavy metal contamination in the FF and negative IVF outcomes [12,16,27]. It should be noted that oocyte quality is influenced not only by the nuclear and mitochondrial genome but also by the microenvironment provided by the ovary and the preovulatory follicle. The oocyte, surrounded by the CCs, is immersed in FF inside the ovarian follicle and is the first to be exposed to environmental contaminants. FF directly provides the microenvironment of granulosa cells and oocytes, and the altered constitution of FF under many conditions can negatively impact oocyte maturation and oocyte quality. In addition, under adverse conditions, the protective effects of CCs may be affected, leading to downstream compromises in steroidogenesis and oocyte development [28].

For these reasons, we assessed if the presence in the FF of HMs, such as Pb and Cd, might interfere with natural fertility and pregnancy rates in ART, affecting CCs, the quality of oocytes, and their functional integrity. 

This paper evaluated for the first time, using transmission electron microscopy study, the possible ultrastructural alterations, Pb- and Cd-dependent, of the human COCs correlated with reproductive outcomes in infertile women. Although the limitation of this study is a small sample size, which may serve as a potential source of bias, we correlated the presence of Pb and Cd levels in the FF of women who were exposed to pollution at the occupational level and undergoing ART with morphological anomalies of both the oocyte and CCs ultrastructure (Group 2) with higher Cd levels (0.26 µg/L–0.41 µg/L). Patients (Group 1) with low levels of Cd (0.10 µg/L–0.25 µg/L) instead had a normal structure of COCs and pregnancies after the ART procedures. Concerning Pb levels in the blood (1.28 µg/dL–3.98 µg/dL) and in the FF (0.49 µg/dL–0.85 µg/dL), these values are lower than minimal health risk levels [22,23,24] in both groups of patients. However, we cannot exclude a synergic effect of both metals present in the FF. 

### 4.1. Oocyte Ultrastructural Changes That Are Heavy Metals Dependent

In oocytes from follicles with higher Cd levels, we found characteristics of cytoplasmic immaturity consisting of an almost complete absence of M-SER aggregates and MV complexes, and of underdeveloped SER (numerous small, dilated SER tubules) not associated with mitochondria, suggesting possible metabolic stress (Cd-dependent) in these cells. 

Many exogenous stressors negatively impact the ER environment and protein processing, and the maturing oocytes are quite sensitive to exogenous stresses. On the other hand, the ER serves many specialized functions in the cell, including calcium storage, biosynthesis of membrane and secretory proteins, and production of phospholipids and sterols. Disturbance of any of these functions can lead to the so-called ER stress.

It is well known that ER is a cellular target of Cd toxicity. Cd increases the cytosolic calcium concentration, inducing calcium release from the ER store. The disruption in ER calcium homeostasis compromises the ER compartment, thus, inducing ER stress [29,30]. Cd potentially blocks calcium signaling at different levels, interfering with its homeostasis and normal protein folding by depleting cell protein sulfhydryl reserves [27]. The effects of the toxicity of Cd on ER stress have been demonstrated in a variety of model organisms such as the yeast *Saccharomyces cerevisiae* [31], primary cultures of cortical neurons [32], porcine pancreatic cells [33], and *Arabidopsis thaliana* [34]. 

The hypothesis of a possible metabolic cell suffering due to ER stress and imbalance of calcium might also explain the presence, in oocytes from follicles with high Cd levels, of a few MV complexes, large communicating dilated SER vesicles transforming into frank vacuoles in the center of ooplasm and pycnotic, or sometimes vacuolated mitochondria in the cortical area. 

It has been demonstrated that ER stress can also induce mitochondrial stress. ER stress triggers increased mitochondrial metabolism, mainly relying on organelle coupling and calcium transfer. In fact, ER and mitochondrial functions are linked via membrane junctions at whose level are concentrated calcium transporters and ion channels. Calcium flux between the two organelles is bi-directionally linked to their functionality. Loss of calcium homeostasis in the endoplasmic reticulum impairs the protein folding machinery, causing an accumulation of unfolded or misfolded proteins in the lumen and resulting in ER dilation and stress. Similarly, impaired mitochondria may cause an accumulation of unfolded and misfolded proteins within their matrix, resulting in stress and mitochondrial collapse [35,36,37,38,39].

In addition, we found mitochondrial matrices containing prominent electron-dense granules. It is known that mitochondrial dysfunction can lead to an abnormal accumulation of these granules [38,40], and a rise in the density, size, and number of dense granules has been reported in a variety of pathological states [38,41]. We hypothesized that altered mitochondria could result from calcium accumulation in the organelle. The presence in oocytes of follicles with high Cd levels of autophagosomes containing mitochondria agrees with the supposed dysfunction of these organelles.

Developing competence to release and respond to calcium is relevant to animal and human IVF programs. At fertilization, intracellular calcium release is crucial for most of the major events that induce oocyte activation and embryonic development. The exocytosis of CGs, a secretory event resulting in the block to polyspermy (cortical reaction), is one of the earliest events depending upon calcium-dependent proteins. In contrast, pre-ovulatory oocytes are incompetent to undergo CG exocytosis due to their inability to release and respond to increases in intracellular calcium [42,43,44]. Particularly, at fertilization/fusion, the inositol 1,4,5-triphosphate (IP3) produced into the ooplasm, in turn, binds to receptors on the endoplasmic reticulum (ER) of the oocyte, causing an oscillatory release of calcium into ooplasm and inducing the fusion of CGs with the oolemma over the entire oocyte’s surface [45]. A zona reaction (hardening of the inner aspect of the ZP) occurs following a cortical reaction [43,46]. In 50% of the oocytes from follicles with high Cd levels, we have observed a ZP with an altered ultrastructure, showing both a non-homogeneous density and a bilayered architecture. The non-homogeneous density characterized by a loose outer area and a dense, compact inner area, associated with a reduction/absence of CGs in the oocyte cortex, can be due to CG exocytosis, which causes inner ZP hardening. So, we hypothesized that stressed ER, Cd-dependent, induces the release of calcium which could determine premature CG exocytosis responsible for the hardening of the inner portion of the ZP.

It has been shown that ovastacin is a core component of CGs required for the post-fertilization removal of sperm-binding sites in the ZP to prevent sperm binding and polyspermy [47]. Our results are in accordance with Zhou et al. (2019) [47], demonstrating that the pouring of ovastacin from CGs into extracellular space before fertilization can cause the hardening of ZP surrounding oocytes and thereby result in the failure of sperm binding and fertilization.

Besides such a specific sign of ZP alteration, we also observed, in the same pool of oocytes subjected to high Cd levels, the presence of a more generic ZP damage represented by delamination of the inner portion of the ZP that led to the formation of a bilayered zona [48]. 

The alteration in ZP consisting of small vesicles and disorganized areas has also been demonstrated by Simoniello et al. (2011) [49] in the oocytes of the female wall lizard Podarcis sicula treated with Cd.

On the other hand, in 50% of oocytes from follicles with high levels of Cd, we found a typical loose microfilamentous architecture of ZP associated with an altered distribution pattern of CG arranged in a discontinuous rim and clustered together. These results also indicate a possible effect of Cd on the cytoskeleton responsible for properly positioning CG and mitochondria in the mature oocyte. Granule and mitochondria migration is a cytoskeleton-dependent process, and microfilaments are required for this cortical translocation in non-mammalian and mammalian models, including humans. The toxicity of Cd on the cytoskeleton was well studied in several animal cells. Exposure to Cd leads to the disassembly of microtubules in Swiss 3T3 cells [50], contributes to depolymerization of the actin cytoskeleton in several cell lines, including rat mesangial cells [44], and Cd-induced actin cytoskeleton alterations and dysfunction of cultured neurons [51]. 

Mitochondrial damage could also be an effect of Cd that has been demonstrated to provoke mitochondrial damage with inhibition of the electron transport chain, reactive oxygen species production, the cytosolic release of pro-apoptotic factors (such as Cyt C), and, finally, activation of the caspase-9 [29]. 

Other authors have demonstrated the adverse effects of Cd on mitochondrial functionality. Dong et al. (2021) [52] found that Cd selectively triggers oxidative stress and mitochondrial injury-mediated apoptosis in trophoblast cells, contributing to placentae impairment and placental-related disorders. Zhou et al. (2019) [47] showed that porcine oocytes that were Cd-exposed suffered impaired cytoplasmatic maturation and that the fertilization capacity of oocytes was disrupted by altered dynamics of mitochondrial integrity and cortical granules. 

It is worth noting that another major mechanism of Cd-induced toxicity is the prolonged generation of reactive oxygen species (ROS) and the changing of intracellular ATP levels [53] that could induce morpho-functional changes in the mitochondria. So, in addition to ER stress and mitochondrial dysfunctionality, oxidative stress also plays a crucial role in Cd-induced toxicity, and ROS have been considered essential mediators for tissue injuries [27]. 

### 4.2. Cumulus Cells Ultrastructural Changes That Are Heavy Metals Dependent

The ultrastructural analysis of CCs shows that in follicles with high Cd levels, these cells do not seem to adhere to each other and are dispersed within a fine and delicate fibrillar extracellular matrix. The capacity to change from a compact cell mass into a dispersed structure of cells during the preovulatory period is a distinctive characteristic of CCs. However, they appear irregularly rounded, polyhedral, or elongated with short and large cytoplasmic processes projecting in the ZP. The shape of nuclei was also altered, changing from oval to flattened and indented. 

In the follicle, CCs play a protective role that critically ensures oocyte competency and may be considered to act as both a bridge and a barrier between the oocyte and the extrafollicular microenvironment. So, CCs are responsible for isolating oocytes from harmful conditions and supporting their needs. CCs defend the oocyte against metabolites, ROS, toxins, and inflammatory markers (cytokines and chemokines) present in the FF. Under adverse conditions, the protective role of CCs may be affected, leading to downstream compromises in steroidogenesis and oocyte development. The intercellular dialog occurs through the gap junctions and paracrine signals. This communication is also important for the same differentiation of CCs. Previous papers have shown that Cd inhibits gap junction intercellular communication and connexin phosphorylation in mouse livers and in normal Balb/3T3 A31 mice [53], and the inhibition of gap junction communication in hepatocytes cannot only protect normal cells but can also aggravate the damage of Cd-exposed cells [54].

Connexin channels are ubiquitous, providing pathways for the movement of molecules between cells (junctional channels) and for the release of molecular effectors into the extracellular environment (plasma membrane hemichannels). To maintain an adequate permeability barrier, hemichannels are tightly regulated by normal extracellular Ca^2+^ to be closed under most conditions [55]. Calmodulin (CaM) is the major calcium sensor in non-muscle cells that binds to calcium, responds to, and regulates intracellular calcium levels, and acts as a common regulator of gap junction communication and hemichannels activity [55,56]. It has also been reported that Cd can displace calcium from CaM, leading to intracellular calcium mobilization [44].

Therefore, we could hypothesize an adverse effect of Cd on intra- and intercellular communication by dysregulation of connexin 43-formed gap junction between CCs and connexin 37-formed gap junction between CCs and oocyte, respectively, inducing a cellular uncoupling [28]. Although properly associated with meiosis resumption, this uncoupling would lead to cytoplasmatic immaturity of the oocyte and the premature expansion of CCs.

The altered cellular morphology of CCs could instead be due to the direct effect of Cd on actin, the major cellular structural protein [44]. 

Moreover, both effects could be the result of a dysregulation of calcium homeostasis that is Cd-dependent.

The assumption of a possible lack of communication between CCs and oocytes could explain the presence in CCs from follicles with high Cd levels, of abundant glycogen granules distributed in the cytoplasm (close to nuclear indentations and around lipid droplets), and of pyknotic and swollen mitochondria in the cytoplasm, and could indicate a possible impairment of the energetic metabolism (of carbohydrates and lipids).

Glucose is significant in every aspect of final oocyte maturation, as demonstrated by its effects on meiotic, cytoplasmic, and cumulus cell maturation, and alterations in glucose metabolism are likely to cause decreased oocyte competence and reduced fecundity. Within the COC, glucose is metabolized via four main pathways, and substrates of these pathways affect oocyte cytoplasmic and nuclear maturation [57].

CCs support energy production in the COC. The oocyte has a poor capacity to utilize glucose and cholesterol synthesis. The CCs metabolize the bulk of the glucose consumed by the COC to supply metabolic intermediates to the oocyte. Particularly, oocytes secrete paracrine signals such as growth differentiation factor 9 (GDF-9) and bone morphogenic protein 15 (BMP-15) necessary to CC’s expansion, differentiation, glycolysis, cholesterol synthesis, and the regulation of cGMP levels. At the same time, CCs provide pyruvate and lactate, products of the cholesterol biosynthetic pathway that are metabolized to produce ATP mainly through oxidative phosphorylation and via the tricarboxylic acid cycle (TCA). So, any metabolic alteration in the somatic follicular cells within COC may affect the oocyte’s development [28,58].

The literature has shown that Cd can potentially limit the glycolysis process in the liver and muscles by inhibiting hexokinase and phosphofructokinase activity [59]. In myocardial cells, Cd exposure induces glucometabolic dysregulation [60]. Therefore, we could assume that in follicles with high Cd levels, CCs, following the suppression of glycolysis, accumulate glucose as glycogen storage. At the same time, the suppression of metabolic shift from aerobic glycolysis to the TCA cycle/ OXOPHOS responsible for the conversion of pyruvate to Acetyl-coenzyme A makes CCs unable to perform steroidogenesis, as demonstrated also by the absence of micro-peroxisomes. These organelles confer steroidogenic ability to these cells.

In the CCs of Group 2 patients, we, moreover, observed the presence of numerous and large lipid droplets, indicating lipid accumulation. Our results are in according to Oluranti et al. (2021), ref. [60] demonstrated the accumulation of lipids (lipotoxicity) in Cd-exposed myocardial cells.

Compared with other tissues, lipid droplets in steroidogenic tissues tend to be smaller in size and more numerous in number precisely because they are thought to be involved in the temporal storage and effective utilization of lipids [61].

Steroid hormones are synthesized de novo from cholesterol in mitochondria and the ER. They are secreted from specialized endocrine cells in the adrenal cortex, testes, and ovaries, and steroidogenic cells have very little steroid hormone storage. For this reason, in these cells, upon stimulation, there is a rapid response to synthesize new steroids, a process that requires a constant supply of cholesterol as a precursor for conversion to steroids. Within steroidogenic tissue, cholesterol is stored in LDs in the form of cholesterol esters (CEs), and the mobilization of these stored CEs is the preferred source of cholesterol for steroidogenesis upon hormone stimulation. The ultrastructure of CCs as steroidogenic elements was described by Nottola et al. (1991) [25]. In fact, the CCs have protidosynthetic capacity and steroid synthetic characteristics, producing small amounts of estrogen and progesterone that can positively change the microenvironment where fertilization will take place [26]. Thus, we think that CCs from follicles with high Cd, in addition to the suppression of metabolic shift from aerobic glycolysis to the TCA cycle/ OXOPHOS Cd, could also inhibit the mobilization of stored CEs necessary for steroids synthesis, resulting in an accumulation of numerous and large lipid droplets. Our observations are in accordance with Knazicka et al. (2014) [62], who demonstrates in human adrenocortical carcinoma cell line NCI-H295R the disruptive effects of Cd, even at very low concentrations, on sexual steroid synthesis, and with Paksy et al. (1999) [63], who found a direct impact in steroid biosynthesis in human ovarian granulosa cells. According to Cd’s endocrine-disrupting activity, the poor steroidogenic activity of CCs might correlate with delayed oocyte cytoplasmic maturation. Finally, we observed cumulus degenerating cells from follicles with high Cd levels characterized by numerous regressing elements such as vacuoles of different sizes containing glycogen granules and involuting organelles, cytoplasmic fragments deriving from cellular blebbing, pyknotic or regressing nuclei, and dense or vacuolized cytoplasm. Thus, even if degenerating, vacuolized, and lysed CCs were observable, most parts of regressing CCs underwent apoptosis. It has been reported that apoptosis can be induced in CCs by oxidative stress and that apoptotic cells were significantly lower in pregnant women than in those who did not become pregnant. In addition, apoptosis-related genes were involved in poor oocyte and embryo development and impaired blastocyst development. So, CC’s apoptosis is related to embryo quality and pregnancy rates, and the degree of granulocyte apoptosis might be inversely associated with the developmental capacity of oocytes [64,65,66]. Moreover, Xu et al. (2021) [67] demonstrated Cd-triggered apoptosis in the human granulosa-like tumor cell line. 

## 5. Conclusions

In this paper, we demonstrated, for the first time, by TEM, important ultrastructural alterations of both oocytes and CCs in human COCs due to the presence of Pb and Cd in the FF of infertile women. 

**Figure 12:**
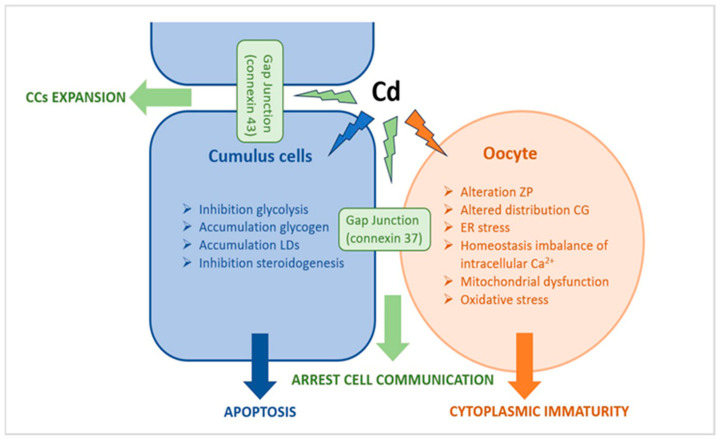
Schematic representation of a possible correlation between ultrastructural alterations of cumulus cell-oocyte complex and dysregulation of different signaling pathways induced by Cd exposure.

We speculated that in COC from follicles with high Cd levels, Cd induces important ultrastructural alterations in both oocyte and CCs. These morphological changes could trigger the maturation arrest of oocyte and the apoptotic death of CCs via multiple effects, including stress ER, homeostasis imbalance of intracellular calcium overload, mitochondrial dysfunction, excessive ROS production, arrest of intra and intercommunication, impairment of energetic metabolism, cellular dysfunction, and apoptosis (Figure 12).

Cd, on the one hand, can have a direct effect on the oocyte, inducing alteration in both ZP texture and the CG’s distribution or exocytosis, together with changes in the morphology of intracytoplasmic organelles (M-SER/MV impairment, ER stress, and mitochondrial alteration) that reduce oocyte quality and reveal cytoplasmic immaturity; on the other hand, Cd can also have an indirect effect on the oocyte, mediated by CCs which become unable to have glycolysis and steroidogenesis, undergo apoptosis, and suffer premature expansion, further affecting the cytoplasmic maturity of the oocyte. 

So, we conclude that Cd influences the oocyte both directly and indirectly (CC-mediated), affecting the same oocyte’s quality, as well as fertilization, early embryonic development, and pregnancy. Although our findings require confirmation due to a limited number of patients, they suggest that even low levels of heavy metals, below or just above the current reference values, significantly affect female reproductive health.

## Figures and Tables

**Table 1:** Clinical characteristics, infertility causes, and IVF outcome of the study patients.

Patients	Age	Infertility Causes	ART Procedure	Collected Oocytes	TransferredEmbryos	Outcome
1	34	Male factor alone	ICSI	6	2	no pregnancy
2	39	unexplained	FIVET	6	3	Pregnancy & miscarriage
3	32	Male factor alone	ICSI	8	3	pregnancy
4	39	Blocked Fallopian tubes	FIVET	9	3	no pregnancy
5	33	Blocked Fallopian tubes	FIVET	13	3	no pregnancy
6	32	Blocked Fallopian tubes	FIVET	11	3	no pregnancy
7	31	Male factor alone	ICSI	9	2	no pregnancy
8	39	Male factor alone	ICSI	10	3	no pregnancy
9	30	Blocked Fallopian tubes	FIVET	15	3	no pregnancy
10	25	Blocked Fallopian tubes	FIVET	12	3	pregnancy
11	29	Male factor alone	ICSI	5	2	No pregnancy
12	30	Blocked Fallopian tubes	FIVET	11	2	No pregnancy
13	37	Blocked Fallopian tubes	FIVET	7	2	No pregnancy
14	24	Blocked Fallopian tubes	FIVET	14	3	pregnancy
15	32	Blocked Fallopian tubes	FIVET	8	2	no pregnancy
16	32	Male factor alone	ICSI	7	3	no pregnancy
17	33	Blocked Fallopian tubes	FIVET	10	3	pregnancy and miscarriage

**Table 2:** Pb and Cd levels in the blood and FF of infertile women were measured using atomic absorption spectrometry. Three/four FF samples were collected from each patient’s individual follicles. Pb values are expressed as µg/dL for Pb and µg/L for Cd. FF values are the means ± SD of 3–4 measurements/patient.

Blood			Follicolar Fluid			
Patients	Mean Pb (µg/dL)	Mean Cd (µg/L)	FF Specimen	Mean Pb (µg/dL) ± SD	Mean Cd (µg/L) ± SD	Groups
1	3.79	1.87	3	0.84 ± 0.098	0. 41 ± 0.017	2
2	2.65	0.90	3	0.67 ± 0.035	0.32 ± 0.021	2
3	1.98	0.74	4	0.61 ± 0.093	0.25 ± 0.015	1
4	2.34	0.73	4	0.64 ± 0.087	0.24 ± 0.009	1
5	2.24	0.78	4	0.66 ± 0.065	0.29 ± 0.007	2
6	3.08	0.80	4	0.72 ± 0.046	0.30 ± 0.009	2
7	2.03	0.72	4	0.59 ± 0.088	0.23 ± 0.016	1
8	3.55	1.69	3	0.85 ± 0.060	0.41 ± 0.012	2
9	2.30	0.59	4	0.65 ± 0.094	0.24 ± 0.014	1
10	1.43	0.60	3	0.49 ± 0.020	0.12 ± 0.007	1
11	2.96	0.63	3	0.62 ± 0.030	0.24 ± 0.013	1
12	3.98	0.77	3	0.78 ± 0.020	0.26 ± 0.015	2
13	3.04	0.65	3	0.72 ± 0.070	0.24 ± 0.012	1
14	1.40	0.54	3	0.53 ± 0.046	0.10 ± 0.008	1
15	2.95	0.80	3	0.71 ± 0.081	0.29 ± 0.013	2
16	2.90	0.60	3	0.68 ± 0.040	0.22 ± 0.013	1
17	1.28	0.54	4	0.55 ± 0.084	0.10 ± 0.009	1

## Data Availability

Not applicable.
